# 抗体偶联药物在非小细胞肺癌中的研究进展

**DOI:** 10.3779/j.issn.1009-3419.2024.102.22

**Published:** 2024-06-20

**Authors:** Yunbo LIU, Sen WEI

**Affiliations:** ^1^300070 天津，天津医科大学（刘畇博）; ^1^Tianjin Medical University, Tianjin 300070, China; ^2^300052 天津，天津医科大学总医院肺部肿瘤外科（韦森）; ^2^Department of Lung Cancer Surgery, Tianjin Medical University General Hospital, Tianjin 300052, China

**Keywords:** 肺肿瘤, 抗体偶联药物, 人表皮生长因子受体2, 人表皮生长因子受体3, 滋养层细胞表面抗原2, Lung neoplasms, Antibody-drug conjugates, Human epidermal growth factor receptor 2, Human epidermal growth factor receptor 3, Trophoblast cell surface antigen 2

## Abstract

肺癌是全球发病率第一位、病死率第二位的恶性肿瘤。非小细胞肺癌（non-small cell lung cancer, NSCLC）是最主要的肺癌病理分型。目前晚期NSCLC的一线标准治疗方案为免疫治疗、靶向治疗，虽然延长了患者的生存期，但获得性耐药仍是不可避免的。抗体偶联药物（antibody-drug conjugates, ADCs）是一类经由连接子将细胞毒性载荷与特异性单克隆抗体偶联制成的新型抗肿瘤药物，与化疗药物相比，ADCs具有精准识别、局部释放、患者耐受性高等优点，近年在NSCLC治疗方面显示出良好的临床获益。本文针对ADCs的作用机制、在晚期NSCLC中的临床研究进展以及存在的问题和挑战等方面进行概述。

## Abstract (en)

Lung cancer is the most common malignant tumor and the second most common malignant tumor in terms of mortality in the world. Non-small cell lung cancer (NSCLC) is the most common pathological type of lung cancer. Currently, the first-line standard treatment for advanced NSCLC is immunotherapy and targeted therapy. Although these treatments prolong the survival of patients, acquired drug resistance is still inevitable. Antibody-drug conjugates (ADCs) are a new type of anti-tumor drug made by coupling cytotoxic payloads to specific monoclonal antibodies via linkers. Compared with chemotherapy drugs, ADCs have the advantages of accurate recognition, local release, and high patient tolerance. In recent years, they have shown good clinical benefits in the treatment of NSCLC. This article provides an overview of the mechanism of action of ADCs, clinical studies progress in advanced NSCLC, and existing problems and challenges.

2020年世界卫生组织国际癌症研究机构公布的数据^[1]^显示，肺癌的发病率居全球第2位，病死率则仍维持在首位。其中非小细胞肺癌（non-small cell lung cancer, NSCLC）约占肺癌的85%，近70%的NSCLC患者在确诊时病理诊断已是晚期，5年生存率不足15%^[2]^。为改善预后，无论是针对驱动基因阴性的转移性NSCLC的化疗与免疫检查点抑制剂联合治疗方案，还是驱动基因阳性的靶向治疗，已成为晚期NSCLC患者治疗的重要选择并显示出临床获益^[3⇓-5]^。然而，免疫治疗、靶向治疗均面临耐药问题。耐药患者在后线治疗中往往具有较高的肿瘤负荷，表现出低应答率及耐受性的下降。为了延长患者的生存时间，积极寻求更优化、可替代的治疗策略迫在眉睫。

抗体偶联药物（antibody-drug conjugates, ADCs）是一类由细胞毒性载荷与单克隆抗体经由连接子偶联组成的新型药物，用于治疗选定的晚期实体肿瘤和血液系统恶性肿瘤。ADCs具有药物强穿透性和抗体-抗原特异性识别两大优势，与常规全身化疗药物相比，可赋予药物对于肿瘤细胞的选择性，具备位点靶向杀伤能力，减少对正常细胞的杀伤，具有减毒增效优势^[6]^。截至2023年12月，全球共有累计17款ADCs已获批用于治疗实体瘤和血液系统肿瘤。ADCs在晚期NSCLC中表现出了较好的疗效，为患者带来了希望。本文就ADCs作用机制及在晚期NSCLC中临床试验研究，总结ADCs的临床疗效及面对的问题并对ADCs进行展望，以期为临床治疗工作的开展提供理论支持。

## 1 ADCs结构与作用机制

ADCs作为一种小分子靶向药物，由单克隆抗体、连接子和有效载荷三个重要部分组成^[6]^。单克隆抗体的构型对应肿瘤组织中有代表性的特异性抗原，人源化或嵌合免疫球蛋白G（immunoglobulin G, IgG）是ADCs最常用的抗体架构^[7]^，具有对靶向抗原的高度亲和力和免疫原性低等特点。理想的抗体靶点是一种细胞表面蛋白，它在肿瘤细胞上过表达，但在健康细胞上低表达或不表达，因此允许选择性杀伤同时伴有较少的全身毒性^[8,9]^。ADCs通过特异性单克隆抗体与肿瘤细胞表面的靶抗原结合形成ADCs-抗原复合物^[10]^。连接子是单克隆抗体与有效载荷的连接桥梁，分为可裂解连接子和不可裂解连接子^[11]^。前者可在肿瘤组织微环境中或靶细胞内迅速解偶联并释放携带的细胞毒性载荷。后者只可依赖靶细胞内的溶酶体降解，完全降解后才能释放细胞毒性载荷^[12]^。两者各有优劣，前者裂解后药物分子量减少便于有效载荷在肿瘤组织细胞和微环境内释放与扩散，不仅直接杀伤肿瘤细胞而且对肿瘤周边组织也产生杀伤效果，即旁观者效应，但也有加剧肿瘤周边正常细胞损伤的风险。后者虽然稳定性强，可减少脱靶毒性的风险，但会影响有效载荷的细胞渗透性从而降低治疗效果^[13]^。有效载荷是ADCs的有效杀伤成分，理想情况下具备低免疫原性和能在低浓度下发挥效果的特性^[14]^，目前搭载的有效载荷多为细胞毒性药物，常为拓扑异构酶抑制剂，如依喜替康衍生物（exatecan derivative, deruxtecan, DXd）、喜树碱衍生物或微管蛋白抑制剂等。有效载荷与抗体的比率被称为抗体比（drug-to-antibody ratio, DAR）：低DAR可降低ADCs的效力，而高DAR则可改变ADCs的药代动力学和毒性^[8]^。

ADCs进入人体后，单克隆抗体与靶抗原特异性结合，触发靶细胞内吞作用使ADCs进入靶细胞内，可裂解连接子在溶酶体蛋白的作用下进行降解（不可裂解连接子无该步骤），细胞毒性药物在细胞内被释放并发挥细胞毒性作用对靶细胞进行杀伤。同时，旁观者效应在杀伤肿瘤组织过程中也起着至关重要的作用。

## 2 ADCs在晚期NSCLC中的研究进展

### 2.1 靶向人表皮生长因子受体2（human epidermal growth factor receptor 2, HER2）的ADCs

 HER2是表皮生长因子受体（epidermal growth factor receptor, EGFR）酪氨酸激酶家族的重要成员^[15,16]^。其可引发受体酪氨酸残基磷酸化，启动丝裂原活化蛋白激酶（mitogen-activated protein kinase, MAPK）、磷脂酰肌醇3-激酶/蛋白激酶B（phosphoinisitide 3-kinase/protein kinase B, PI3K/Akt）等信号通路，促进癌症发生^[17]^。HER2在NSCLC中的变异按程度分为突变（2%-4%）、扩增（1%-4%）和过表达（20%）三类^[18,19]^。本段落重点讨论针对HER2阳性NSCLC的2个ADCs，即Trastuzumab deruxtecan（T-DXd）和Trastuzumab emtansine（T-DM1）。

T-DXd是靶向HER2阳性晚期NSCLC的新型ADCs，由抗HER2抗体（曲妥珠单抗）、四肽基类可切割连接子和拓扑异构酶I抑制剂（Deruxtecan）细胞毒性药物组成^[20,21]^。相较于第一代T-DM1，T-DXd的整体构型使其具有更强的组织穿透力和较高的DAR（T-DXd为8，T-DM1为3.5）^[22]^，对肿瘤靶细胞杀伤力强及充分的旁观者效应能杀伤肿瘤病灶。T-DXd也获得了美国食品药品监督管理局（Food and Drug Administration, FDA）的加速批准，截止于2024年1月1日正在进行的临床试验包括：DESTINY-Lung01（NCT03505710）^[23]^、DESTINY-Lung02（NCT04644237）^[24]^、DESTINY-Lung03（NCT04686305）^[25]^以及DESTINY-Lung04（NCT05048797）^[26]^。本文着重归纳总结已发布的成果。

DESTINY-Lung01是一项多中心、开放标签、临床II期研究^[23]^。对既往铂类化疗后HER2突变或HER2过表达的晚期NSCLC患者进行临床试验，探究T-DXd对非鳞状NSCLC的疗效及肿瘤组织中HER2表达水平对T-DXd响应的影响。筛选入组HER2突变患者91例，接受6.4 mg/kg剂量3周治疗方案。观察临床疗效、不良事件和药物相关间质性肺病（drug-induced interstitial lung disease, DILD）发生率。研究显示，HER2突变组（91例）中位随访时间为13.1个月，客观缓解率（objective response rate, ORR）为55%（95%CI: 44%-65%）；中位缓解持续时间（median duration of response, mDOR）为9.3个月（95%CI: 5.7-14.7）；中位无进展生存期（median progression-free survival, mPFS）为8.2个月（95%CI: 6.0-11.9），中位总生存期（median overall survival, mOS）为17.8个月（95%CI: 13.8-22.1）。不良反应主要表现为中性粒细胞减少、贫血等血液毒性反应，26%的患者发生显著的药物剂量相关毒性反应，如DILD，并导致2例患者死亡^[23]^。详见表1。临床试验证实T-DXd 6.4 mg/kg剂量治疗HER2突变型晚期NSCLC，具有较好的临床疗效、持久的抗癌活性和可控的安全性；试验结果也提示在临床试验过程中医务人员应密切监测DILD的发生，需在发生早期干预调整药物用药方案。试验同时发现HER2表达水平不影响T-DXd药物出现响应现象，这一成果有可能扩展该药物的适用患者群，但因该试验样本量有限，还需要在以后的研究中扩大样本量、扩充筛选标准进行进一步探索。试验也存在一定局限性，T-DXd用于乳腺癌的临床试验Destination-Breast01成果确定推荐剂量为：5.4、6.4 mg/kg^[27]^。DESTINY-Lung01试验仅证实T-DXd 6.4 mg/kg剂量模式具有抗肿瘤疗效，但未对5.4 mg/kg剂量模式治疗效果进行研究。

**表1:** DESTINY-Lung01研究数据

Drugs	Clinical curative effect						Drug-related adverse events				DILD
	ORR	mPFS (mon)	mDOR (mon)	mOS (mon)	DCR		Grade 1-2	Grade 3-4	Grade 5	Common symptoms	
T-DXd	55% (95%CI: 44%-65%)	8.2 (95%CI: 6.0-11.9)	9.3 (95%CI: 5.7-14.7)	17.8 (95%CI: 13.8-22.1)	92% (95%CI: 85%-97%)		20%	46%	14%	Neutropenia (19%) and anemia (10%)	26% (24/92)
Pyrotinib	30% (95%CI: 18.8%-43.2%)	6.9 (95%CI: 5.5-8.3)	6.9 (95%CI: 4.9-11.1)	14.4 (95%CI: 12.3-21.3）	66%		-	-	-	Diarrhea (88.3%) and elevated creatinine (28.3%)	-
Poziotinib	27.8% (95%CI: 18.9%-38.2%)	5.5 (95%CI: 0.03-13.1)	5.1 (95%CI: 1-12.3)	-	70%		-	-	-	Rash (42%), mucositis (23%)	-

ORR: objective response rate; mPFS: median progression-free survival; mDOR: median duration of response; mOS: median overall survival; DCR: disease control rate; DILD: drug-induced interstitial lung disease.

基于DESTINY-Lung01临床试验成果及提出的问题，盲法、多中心、II期临床研究DESTINY-Lung02试验正式启动^[24]^，研究T-DXd 5.4 mg/kg的剂量模式对HER2突变型转移性NSCLC的临床疗效，并对比6.4 mg/kg剂量模式。试验共有152例转移性NSCLC患者入组，分为5.4和6.4 mg/kg两个剂量组。临床试验结果见表2，5.4、6.4 mg/kg剂量组ORR分别为49.0%（95%CI: 39.0%-59.1%）和56.0%（95%CI: 41.3%-70.0%）。采用REVEL试验的传统Ramucirumab+Docetaxel治疗方案作为基准对照组^[28]^，其ORR仅为22.9%，T-DXd药物治疗HER2突变型转移性NSCLC疗效有较大提升。同时5.4和6.4 mg/kg两个剂量组PFS分别为19.5个月和未予评估（not evaluated, NE）。预估12个月的OS率分别为67%和73%，显示该试验均具有持久的抗肿瘤效果。药物相关不良事件分别为97例（96.0%）和50例（100%）；两组DILD发生率分别为5.9%和8.0%。两种剂量对治疗转移性NSCLC的安全性是可以接受的，总体上对肿瘤进展具有相近的控制能力。试验结果充分证实5.4 mg/kg剂量组的用药安全性高于6.4 mg/kg剂量组。有理由认为5.4 mg/kg T-DXd剂量方案有潜力作为HER2突变的晚期NSCLC人群的标准治疗方案。

**表2:** DESTINY-Lung02研究数据

Index		DESTINY-Lung02	
Experimental design			
Drugs		T-DXd	T-DXd
Research object		HER2 mutant-NSCLC	HER2 mutant-NSCLC
Drug dose		5.4 mg/kg	6.4 mg/kg
Drug administration frequency		Once every 3 weeks	Once every 3 weeks
Number of members enrolled (2:1)		101	50
Experimental results			
Clinical curative effect	ORR	49.0% (95%CI: 39.0%-59.1%)	56.0% (95%CI: 41.3%-70.0%)
	CR	1 (1.0%)	2 (4.0%)
	PR	49 (48.0%)	26 (52.0%)
	mPFS	9.9 mon (95%CI: 7.4-NE)	15.4 mon (95%CI: 8.3-NE)
	Estimated OS rate in December	67.0% (95%CI: 56%-76%)	73.0% (95%CI: 57%-84%)
Drug-related AEs	Total	96.0% (97/101)	100.0% (50/50)
	Grade≥3	38.6% (95%CI: 29.1%-48.8%)	58.0% (95%CI: 43.2%-71.8%)
	Drug-related AEs	Nausea (67.3%), neutropenia (61.4%), anemia (10.9%)	Nausea (82.0%), neutropenia (92.0%), anemia (16.0%)
	Drug withdrawal caused by AEs	13.9%	20.0%
	Reduced dose caused by AEs	16.8%	32.0%
	Temporary interruption caused by AEs	26.7%	48.0%
Incidence of DILD		5.9%	8.0%

HER2: human epidermal growth factor receptor 2; NSCLC: non-small cell lung cancer; CR: complete response; PR: partial response; AEs: adverse events; NE: not evaluated.

同期进行中的研究还有探究T-DXd与铂类-培美曲塞-度伐利尤单抗方案临床疗效优劣的DESTINY-Lung03（NCT04686305）试验^[25]^，该试验旨在评估T-DXd与免疫治疗药物以及化疗药物单独或联合使用的安全性、耐受性以及有效性；以及研究T-DXd与铂类-培美曲塞-帕博利珠单抗方案临床疗效优劣的DESTINY-Lung04（NCT04686305）试验^[26]^。以上两试验目前还在研究进展阶段，尚未获得试验数据。

T-DM1是靶向HER2阳性晚期NSCLC的ADCs，将曲妥珠单抗的HER2靶向作用与微管抑制剂美坦辛DM1结合起来。T-DM1与HER2阳性肿瘤细胞结合，通过受体介导的内吞作用进入细胞内，经由溶酶体降解释放出有效载荷DM1^[21]^。T-DM1为HER2阳性晚期NSCLC患者提供了新的可选治疗方法。NCT02675829是一项T-DM1针对HER2阳性晚期NSCLC患者的II期临床研究试验^[29]^，结果显示PFS为5个月，ORR为51%，HER2扩增、突变和共突变/扩增患者的应答率分别55%、50%和50%。该研究获得了令人鼓舞的结果，接受治疗者展现出了良好的疗效，同时高耐受性使得不良反应相对较少，主要为贫血和血小板减少。基于此，美国国立综合癌症网络（National Comprehensive Cancer Network, NCCN）推荐T-DM1为治疗HER2阳性晚期NSCLC的2A类药物，但其在临床使用过程中也暴露出受肿瘤细胞HER2表达水平影响较大的缺点^[30]^。期待NCT02675829、NCT03784599等疗效和安全性相关的II期临床研究得出结果。

### 2.2 靶向人表皮生长因子受体3（human epidermal growth factor receptor 3, HER3）的ADC

HER3作为EGFR家族的重要成员，尽管本身不具备激酶活性，却能够与家族内的其他成员结合，形成同源或异源二聚体。这种结合促使受体酪氨酸残基发生磷酸化，进而激活PI3K/Akt、MAPK等关键信号通路，最终导致癌症的发生和发展^[31]^。研究^[32]^显示，19%的NSCLC存在HER3过表达，HER3在肿瘤细胞上的过表达被视为NSCLC对抗EGFR-酪氨酸激酶抑制剂（EGFR-tyrosine kinase inhibitors, EGFR-TKIs）产生耐药性的关键机制之一。

Patritumab deruxtecan（也称为HER3-DXd或U3-1402）是一种针对NSCLC的HER3靶向新型ADCs，由四肽基可裂解连接子偶联人免疫球蛋白G1单克隆抗体和拓扑异构酶I抑制剂细胞毒性药物构成^[33]^。可裂解连接子可在肿瘤微环境中裂解，发挥旁观者效应^[34]^。尽管T-DXd使用相同的连接-有效载荷系统，但与抗HER2单克隆抗体类相比，它具有较低的结合水平和更高的内化率。其促凋亡作用主要来自于其有效载荷的细胞毒性，而不是抑制HER3/PI3K/Akt通路^[33]^。HER3-DXd作为NSCLC的HER3位点靶向ADCs临床试验为HERTHENA-Lung01。

HERTHENA-Lung01包括I期临床试验（也称为U31402-A-U102）和后续的II期临床试验。U31402-A-U102探究HER3-DXd对携带EGFR突变并既往经受EGFR-TKIs治疗的晚期或转移性NSCLC的临床疗效。分剂量递增/扩增两个阶段队列试验研究，剂量递增阶段招募36例对至少一种EGFR-TKIs具有耐药性的患者，分为4个剂量组，以连续重新评估方法为指导，基于剂量限制性毒性（dose-limiting toxicity, DLT）开展，5.6 mg/kg组的终点ORR为四组中最高，因此确定HER3-DXd的推荐剂量为5.6 mg/kg。扩增剂量队列研究在5.6 mg/kg剂量组继续纳入45例患者，合并递增阶段患者后共计57例接受了HER3-DXd 5.6 mg/kg治疗，ORR为39%（95%CI: 26.0%-52.4%），优于传统化疗药物^[35]^。中位客观缓解时间（median objective response, mOR）为2.6个月，DOR为6.9个月。至少有一项可评估肿瘤变化患者51例，其中27例（53%）接受HER3-DXd治疗后，影像学可见肿瘤总直径减小≥30%，疗效显著。该试验报告的不良事件主要为血小板和中性粒细胞减少等血液学毒性事件。试验证实：HER3-DXd 5.6 mg/kg剂量方案具有较为可控的安全性，HER3-DXd治疗携带EGFR突变和晚期或转移性NSCLC的临床试验研究获得成功。

后续的HERTHENA-Lung01 II期试验是一项多中心、双队列临床研究，探究HER3-DXd对既往接受过EGFR-TKIs且伴有EGFR突变的晚期NSCLC临床疗效^[36]^。结果显示：ORR为29.8%（95%CI: 23.9%-36.2%）；mDOR为6.4个月，43.3%的患者反应持续时间≥6个月。mPFS为5.5个月。210例患者在基线治疗后肿瘤出现可观察到的影像学改变，出现不同程度缩小。

两期临床试验^[35,36]^总计有64.9%和28.9%的患者发生≥3级药物相关不良事件。不良事件主要为血小板减少和中性粒细胞减少。12例（5.3%）患者诊断DILD。同时结果显示，接受过免疫治疗的患者药物相关不良事件发生率高于未接受免疫治疗的患者。DILD的发生率（8%）也高于未接受免疫治疗患者（4%）。这一数据虽没有统计学意义但也引起了研究者的关注。

综上所述，针对EGFR-TKIs和铂类化疗失败后的EGFR突变晚期NSCLC患者，HER3-DXd 5.6 mg/kg剂量方案具有可控安全性的同时显示出较好的临床疗效。对第三代EGFR-TKIs耐药及不适合第三代EGFR-TKIs的患者来说，有可能是一种新的治疗方案。

HERTHENA-Lung02（NCT05338970）^[37]^观察HER3-DXd对经EGFR-TKIs治疗后肿瘤组织出现进一步进展的EGFR突变晚期NSCLC患者的临床疗效。为了提升治疗效果，联合疗法显得尤为重要。Yonesaka等研究^[34]^表明，针对EGFR突变的TKIs与HER3-DXd联合应用能显著增强对EGFR突变NSCLC患者的疗效，其潜在机制可能涉及EGFR-TKIs上调肿瘤细胞表面HER3的表达水平。此外，HER3-DXd与EGFR-TKIs的联合使用可能增强HER3-DXd的抗癌作用。同时，Haratani等^[38]^进行的程序性死亡受体1（programmed cell death 1, PD-1）与HER3-DXd的联合用药临床研究显示，这种组合能激发更强烈的免疫反应并展现出更优的抗肿瘤效果。

### 2.3 靶向滋养层细胞表面抗原2（trophoblast cell surface antigen 2, TROP2）的ADCs

TROP2是一种可介导肿瘤生长和转移的跨膜糖蛋白钙信号转导子^[39]^。TROP2在75%的肺鳞癌、64%的肺腺癌和18%的肺高级别神经内分泌癌中有高表达，是一个预后较差的特征^[40]^。

Datopotamab deruxtecan（Dato-DXd, DS-1062）是一种新型的TROP2靶向ADCs，由四肽可裂解连接子将人源化的抗TROP2免疫球蛋白1单克隆抗体和拓扑异构酶I抑制剂（DXd）共价连接。该药物目前尚处于临床I期试验TROPION-PanTumor01。该研究的队列数据已多次在各大国际学术会议如美国临床肿瘤学会、世界肺癌大会、欧洲肿瘤内科学会等进行公示。

TROPION-PanTumor01是一项正在美国和日本的17个地点进行的I期、递增/扩增两阶段、多中心的临床队列试验^[41]^。第一阶段的主要目的是随访患者初步判断Dato-DXd安全性和患者耐受性，为第二阶段试验确定最大耐受剂量（maximum tolerated dose, MTD）和用药剂量提供依据。按入组顺序递增药物剂量评估^[42,43]^限制毒性反应≥3例时终止该阶段试验。临床试验结果见表3，结果分析MTD为8 mg/kg。150例接受不同剂量Dato-DXd的NSCLC患者中，4.0、6.0、8.0 mg/kg组确认的ORR分别为22.0%、26.0%和23.8%，疾病控制率（disease control rate, DCR）分别为76.0%、70.0%和78.8%；药物相关不良反应在4、6和8 mg/kg之间呈现出剂量依赖性，包括呕吐（7% vs 9% vs 29%）、贫血（4% vs 11% vs 22%）、腹泻（5% vs 9% vs 15%）和黏膜炎（6% vs 6% vs 16%）。与4 mg/kg组（10%）相比，8 mg/kg组（36%）的严重药物相关不良反应发生率要高3倍，同时4.0、6.0、8.0 mg/kg组DILD发生率分别为10.0%、6.0%和13.8%^[41]^。综合临床试验公布的临床疗效和不良事件数据，该试验将6 mg/kg定为Dato-DXd的临床治疗应用推荐剂量。分别评估Dato-DXd联合Pembrolizumab和Durvalumab在晚期NSCLC中疗效的I期临床试验TROPION-Lung02和TROPION-Lung04正在进行中，现有数据表明Dato-DXd在包括晚期NSCLC在内的早期临床试验中显示出了良好的活性^[44]^。

**表3:** TROPION-PanTumor01剂量扩增队列研究数据

Index		Dose amplification cohort of TROPION-PanTumor01		
Experimental design				
Drugs		Dato-DXd	Dato-DXd	Dato-DXd
Research object		TROP2 mutant-NSCLC	TROP2 mutant-NSCLC	TROP2 mutant-NSCLC
Drug dose		4 mg/kg	6 mg/kg	8 mg/kg
Drug administration frequency		Once every 3 weeks	Once every 3 weeks	Once every 3 weeks
Number of members enrolled (1:1:1.6)		50	50	80
Experimental results				
Clinical curative effect	ORR	22.0% (95%CI: 11.5%-36.0%)	26.0% (95%CI: 14.6%-40.3%)	23.8% (95%CI: 14.9%-34.6%)
	DCR	76.0% (95%CI: 61.8%-86.9%)	70.0% (95%CI: 55.4%-82.1%)	78.8% (95%CI: 68.2%-87.1%)
	DOR (mon)	12.7 (95%CI: 2.8-NE)	10.5 (95%CI: 5.6-26.5)	9.6 (95%CI: 5.8-NE)
	PFS (mon)	4.3 (95%CI: 2.9-6.9)	6.9 (95%CI: 2.7-8.8)	5.2 (95%CI: 4.1-7.1)
	OS (mon)	12.9 (95%CI: 9.4-NE)	11.4 (95%CI: 7.1-20.6)	10.5 (95%CI: 8.0-12.0)
Drug-related AEs	Total	94.0% (47/50)	82.0% (41/50)	97.5% (78/80)
	Grade 1-2	80.0% (40/50)	56.0% (28/50)	61.3% (49/80)
	Grade≥3	14.0% (7/50)	26.0% (13/50)	36.3% (29/80)
	Drug withdrawal caused by AEs	16.0% (8/50)	14.0% (7/50)	23.8% (19/80)
	Reduced dose caused by AEs	2.0% (1/50)	10.0% (5/50)	27.5% (22/80)
Incidence of DILD		10.0% (5/50)	6.0% (3/50)	13.8% (11/80)

TROP2: trophoblast cell surface antigen 2.

### 2.4 临床试验状态中的ADCs研究

除以上详述的靶点药物外目前还有几种ADCs仍处于试验状态：（1）TROP2受体靶向的Sacituzumab govitecan，该药物已获批用于乳腺癌和前列腺癌的治疗，其目前针对晚期NSCLC的Ib期临床试验编码为NCT01631552，已公开数据显示ORR为17.7%（95%CI: 9.2%-29.5%），不良事件以中性粒细胞减少为主^[45]^；（2）c-MET受体靶向的Telisotuzum‐ab Vedotin（Teliso-V），目前进行中的I期临床试验编码为NCT02099058，该试验研究Teliso-V联合Nivolumab治疗晚期NSCLC的安全性和患者耐受性，其次是抗肿瘤活性，公开数据显示ORR为19%（95%CI: 14%-26%），不良事件以中性粒细胞减少、贫血和低蛋白血症为主^[46]^，项目总结详见表4。

**表4:** NSCLC ADC临床试验总结

ADC	Target point	NCT code	Research stage	Primary end-point		Drug-related AEs (Grade≥3)		DILD
						Proportion	Major drug-related AEs	
Trastuzumab deruxtecan (T-DXd)	HER2	NCT03505710 (DESTINY-Lung01)	II	ORR (50/91)		46%	Neutropenia (19%) and anemia (10%)	0.26%
		NCT04644237 (DESTINY-Lung02)	II	ORR	49% (5.4 mg/kg)	38.6%	Neutropenia (18.8%), anemia (10.9%)	5.9%
					56% (6.4 mg/kg)	58.0%	Neutropenia (36.0%), anemia (16.0%)	8.0%
		NCT04686305 (DESTINY-Lung03)	Ib	Incidence of AEs		-	-	-
		NCT05048797 (DESTINY-Lung04)	III	PFS		-	-	-
Trastuzumab emtansine (T-DM1)	HER2	NCT02675829	II	ORR	44.4% (8/18)	-	-	-
					52.1% (25/48)	-	-	-
Patritumab deruxtecan	HER3	NCT03260491	I	ORR	25%	-	Thrombocytopenia (28%), neutropenia (19%), fatigue (9%)	5.2%
		NCT04619004	II	ORR	29.8%	64.9%	Hematological toxicity, followed by thrombocytopenia and neutropenia	5.3%
Sacituzumab govitecan	TROP2	NCT01631552	Ib	ORR	17.7%		Neutropenia (28%), diarrhea (7%), agranulocytosis with fever (4%)	
Datopotamab deruxtecan (Dato-DXd)	TROP2	NCT03401385	I	ORR	22.0% (4 mg/kg)	14.0%	Nausea, stomatitis, fatigue, vomiting, loss of appetite, constipation, infusion-related reactions, interstitial pneumonia	8.0% (4/50)
					26.0% (6 mg/kg)	26.0%		6.0% (3/50)
					23.8% (8 mg/kg)	36.6%		12.5% (10/80)
Telisotuzumab vedotin	C-MET	NCT02099058	I	ORR	19%	-	Neutropenia, Anemia, Hypoproteinemia	-

## 3 ADCs治疗NSCLC临床试验暴露问题分析

### 3.1 ADCs潜在耐药的风险

根据ADCs作用机制的研究，药物进入体循环至发挥作用的过程中其在体循环中的存在形态主要为3种：未裂解的ADCs、游离的抗体成分以及被释放的细胞毒性药物^[47]^。ADCs潜在的耐药环节众多，耐药机制尚未得到充分论证。可能的耐药机制为：（1）靶抗原表达的改变，ADCs的聚集和裂解依赖于单克隆抗体与靶抗原结合。如果肿瘤细胞的靶抗原表达降低或完全丧失，ADCs将失去治疗效果；（2）免疫逃逸，肿瘤细胞可能通过改变其表面的抗原表达种类或调节表面抗原的表达数量，逃避ADCs的识别和攻击；（3）肿瘤微环境的变化，肿瘤周围的微环境会影响ADCs的聚集、识别、内吞等步骤，微环境中存在的酶等物质增多也可能会提前分解ADCs，或者通过提供保护性因子帮助肿瘤细胞抵抗ADCs的攻击；（4）基因突变，在ADCs治疗过程中，肿瘤细胞可能会经历基因突变，产生耐药的细胞亚群；（5）旁路途径的激活，肿瘤细胞可能会激活旁路途径来躲避ADCs所阻断的关键信号通路从而减弱有效载荷的杀伤效果等^[48,49]^。由于ADCs在NSCLC中的临床试验研究较其他实体瘤临床研究时间较短，其耐药机制还有待后续临床试验的进一步论证。

### 3.2 ADCs存在的毒副作用

上述ADCs临床试验结果显示，各类各期临床试验中出现的药物相关不良事件主要表现为呕吐等胃肠道反应和血液系统毒性反应。分析原因，ADCs相关不良事件的成因与其作用机制紧密关联，如体内表达靶抗原的正常细胞会被异常识别造成非目标性杀伤；连接子在非肿瘤组织环境中提前裂解释放有效载荷发生脱靶毒性等^[8]^。在明确ADCs相关不良事件种类的同时也需要总结临床处理经验，完善相应的用药安全评估机制和不良事件处理原则指南。临床试验过程中另一类不良事件为DILD，应引起研究者的重视，因为已有研究因永久DILD而停止药物临床试验，更严重出现DILD相关死亡病例。截止目前虽然DILD发病机制尚未有研究证实，但Kumagai等^[50]^通过临床试验推断可能机制为：（1）癌细胞靶向依赖性摄取致ADCs在癌细胞周边高浓度聚集；（2）健康肺细胞对ADCs的靶向非依赖性摄取导致正常细胞被误杀伤风险增高；（3）癌细胞释放的有效载荷的旁观者杀伤效应；（4）ADCs提前裂解导致有效载荷进入血液循环，肺组织损伤风险高。对DILD的治疗目的是抑制炎症和防止纤维化组织的形成，治疗的基石是及时使用皮质类固醇治疗。依据抗肿瘤DILD诊治专家共识推荐^[51]^，在ADCs应用过程中密切监测咳嗽、呼吸困难和发热症状，观察到DILD时应立刻停止用药，对于无症状并通过肺X线检查确诊的1级DILD患者，只有症状完全缓解后才能重新开始用药，并在28 d内进行随访胸部影像学检查，以确定可否恢复剂量或较长时间中断治疗。如果在停用药物的情况下DILD仍出现进展（2-4级），则建议患者立即接受皮质类固醇治疗。

## 4 展望

综上所述，ADCs在晚期NSCLC特别是既往抗肿瘤治疗过程发生耐药的NSCLC中展示出了较好治疗效果及治疗安全性，但为达成主要目标，即最大限度地提高抗肿瘤活性，同时限制靶上和靶外毒性，依然还有继续努力、探索的需求。

ADCs是治疗晚期NSCLC的新策略，HER2、HER3和TROP2等几种靶向药物在早期临床试验中显示出了成功的临床效果，伴随ADCs研发领域更多技术攻关、技术创新，能更好地实现ADCs高效、低毒、精准的设计目标；探索ADCs与其他药物或治疗方法联合治疗策略，进一步提高ADCs的疗效，增强抗肿瘤免疫反应和对抗耐药性，以便为广大晚期NSCLC患者带来福音。
